# Supplementary material for: Genome-wide cross-disease analyses highlight causality and shared biological pathways of type 2 diabetes with gastrointestinal disorders
Source: Commun Biol. 2024 May 27;7:643. doi: 10.1038/s42003-024-06333-z (PMC11130317; doi:10.1038/s42003-024-06333-z)
Supplement: Supplementary file 2 — Description of Additional Supplementary Files.docx [file 42003_2024_6333_MOESM2_ESM.docx]

Description of Additional Supplementary Files

File name: Supplementary Data I

Description: Comprehensive information on the GWAS datasets analysed in this study.

File name: Supplementary Data 2

Description: Presentation of the global genetic correlation between:

- type 2 diabetes and GI disorders (with and without the MHC region)
- BMI-adjusted T2D and GI disorders
- and across GI disorders

File name: Supplementary Data 3

Description: Presentation of comprehensive results of local genetic correlations between type 2 Diabetes (T2D) and gastrointestinal (GI) disorders.

File name: Supplementary Data 4

Description: Full results of the Mendelian randomisation (MR) analysis between type 2 diabetes and gastrointestinal disorders

File name: Supplementary Data 5

Description: Genome-wide significant (sentinel) genes identified in our study for type 2 diabetes.

File name: Supplementary Data 6

Description: Genome-wide significant (sentinel) genes identified in our study for inflammatory bowel disease.

File name: Supplementary Data 7

Description: Genome-wide significant (sentinel) genes identified in our study for diverticular disease.

File name: Supplementary Data 8

Description: Genome-wide significant (sentinel) genes identified in our study for gastroesophageal reflux disease.

File name: Supplementary Data 9

Description: Genome-wide significant (sentinel) genes identified in our study for peptic ulcer disease.

File name: Supplementary Data 10

Description: Genome-wide significant (sentinel) genes identified in our study for gastritis-duodenitis.

File name: Supplementary Data 11

Description: Genome-wide significant (sentinel) genes identified in our study for irritable bowel syndrome.

File name: Supplementary Data 12

Description: Genome-wide significant (sentinel) genes shared by type 2 diabetes and inflammatory bowel disease.

File name: Supplementary Data 13

Description: Genome-wide significant (sentinel) genes shared by type 2 diabetes and diverticular disease.

File name: Supplementary Data 14

Description: Genome-wide significant (sentinel) genes shared by T2D and GI disorders (IBS, GERD, and PUD).

File name: Supplementary Data 15

Description: Genome-wide significant (sentinel) genes shared by T2D and GI disorders (IBD, diverticular disease, and gastritis-duodenitis).

File name: Supplementary Data 16

Description: Genome-wide significant (sentinel) T2D genes shared by peptic ulcer disease.

File name: Supplementary Data 17

Description: Genome-wide significant (sentinel) T2D genes shared by gastroesophageal reflux disease.

File name: Supplementary Data 18

Description: Genome-wide significant (sentinel) T2D genes shared by gastritis-duodenitis.

File name: Supplementary Data 19

Description: Genome-wide significant (sentinel) T2D genes shared by diverticular disease.

File name: Supplementary Data 20

Description: Genome-wide significant (sentinel) T2D genes shared by irritable bowel syndrome.

File name: Supplementary Data 21

Description: Genome-wide significant (sentinel) T2D genes shared by inflammatory bowel disease.

File name: Supplementary Data 22

Description: Genome-wide significant (sentinel) PUD genes shared by T2D.

File name: Supplementary Data 23

Description: Genome-wide significant (sentinel) GERD genes shared by T2D.

File name: Supplementary Data 24

Description: Genome-wide significant (sentinel) gastritis-duodenitis genes shared by T2D.

File name: Supplementary Data 25

Description: Genome-wide significant (sentinel) diverticular disease genes shared by T2D.

File name: Supplementary Data 26

Description: Genome-wide significant (sentinel) irritable bowel syndrome genes shared by T2D.

File name: Supplementary Data 27

Description: Genome-wide significant (sentinel) inflammatory bowel disease genes shared by T2D.

File name: Supplementary Data 28

Description: Genes reaching genome-wide significance in the FCP analysis for T2D and PUD.

File name: Supplementary Data 29

Description: Genes reaching genome-wide significance in the FCP analysis for T2D and GERD.

File name: Supplementary Data 30

Description: Genes reaching genome-wide significance in the FCP analysis for T2D and gastritis-duodenitis.

File name: Supplementary Data 31

Description: Genes reaching genome-wide significance in the FCP analysis for T2D and diverticular disease.

File name: Supplementary Data 32

Description: Genes reaching genome-wide significance in the FCP analysis for T2D and irritable bowel syndrome.

File name: Supplementary Data 33

Description: Genes reaching genome-wide significance in the FCP analysis for T2D and inflammatory bowel disease.

File name: Supplementary Data 34

Description: Genes reaching genome-wide significance in the FCP analysis shared across T2D, gastritis-duodenitis and peptic ulcer disease.

File name: Supplementary Data 35

Description: Genes reaching genome-wide significance in the FCP analysis shared across T2D, gastroesophageal reflux disease and peptic ulcer disease.

File name: Supplementary Data 36

Description: Genes reaching genome-wide significance in the FCP analysis shared across T2D, diverticular disease and peptic ulcer disease.

File name: Supplementary Data 37

Description: Genes reaching genome-wide significance in the FCP analysis shared across T2D, GERD, diverticular disease and peptic ulcer disease.

File name: Supplementary Data 38

Description: Genes reaching genome-wide significance in the FCP analysis shared across T2D, GERD, diverticular disease, IBD, IBS, and gastritis-duodenitis.

File name: Supplementary Data 39

Description: Genes reaching genome-wide significance in the FCP analysis shared across T2D, GERD, diverticular disease, IBD, and IBS.

File name: Supplementary Data 40

Description: Genes overlapping T2D_bmiadj_ and GERD at P_gene_ < 0.05.

File name: Supplementary Data 41

Description: Genes overlapping T2D_bmiadj_ and GERD at P_gene_ < 0.05 with the MHC region excluded.

File name: Supplementary Data 42

Description: Genes overlapping T2D_bmiadj_ and IBS at P_gene_ < 0.05.

File name: Supplementary Data 43

Description: Genes overlapping T2D_bmiadj_ and IBS at P_gene_ < 0.05 with the MHC region excluded.

File name: Supplementary Data 44

Description: Genes overlapping T2D_bmiadj_ and gastritis-duodenitis at P_gene_ < 0.05.

File name: Supplementary Data 45

Description: Genes overlapping T2D_bmiadj_ and gastritis-duodenitis at P_gene_ < 0.05 with the MHC region excluded.

File name: Supplementary Data 46

Description: Genes overlapping T2D_bmiadj_ and peptic ulcer disease at P_gene_ < 0.05.

File name: Supplementary Data 47

Description: Genes overlapping T2D_bmiadj_ and peptic ulcer disease at P_gene_ < 0.05 with the MHC region excluded.

File name: Supplementary Data 48

Description: Genes overlapping T2D_bmiadj_ and diverticular disease at P_gene_ < 0.05.

File name: Supplementary Data 49

Description: Genes overlapping T2D_bmiadj_ and diverticular disease at P_gene_ < 0.05 with the MHC region excluded.

File name: Supplementary Data 50

Description: Genes overlapping T2D_bmiadj_ and inflammatory bowel disease at P_gene_ < 0.05.

File name: Supplementary Data 51

Description: Genes overlapping T2D_bmiadj_ and inflammatory bowel disease at P_gene_ < 0.05 with the MHC region excluded.

File name: Supplementary Data 52

Description: Biological pathways significantly enriched for genes overlapping type 2 diabetes and gastroesophageal reflux disease.

File name: Supplementary Data 53

Description: Biological pathways significantly enriched for genes overlapping type 2 diabetes and irritable bowel syndrome.

File name: Supplementary Data 54

Description: Biological pathways significantly enriched for genes overlapping type 2 diabetes and gastritis-duodenitis.

File name: Supplementary Data 55

Description: Biological pathways significantly enriched for genes overlapping type 2 diabetes and peptic ulcer disease.

File name: Supplementary Data 56

Description: Biological pathways significantly enriched for genes overlapping type 2 diabetes and diverticular disease.

File name: Supplementary Data 57

Description: Biological pathways significantly enriched for genes overlapping type 2 diabetes and inflammatory bowel disease.
